# Sternal reconstruction using locking compression plates (LCP): our experience in Egypt, a case series

**DOI:** 10.1186/s13019-020-01266-0

**Published:** 2020-08-21

**Authors:** Kareem Ahmed, Mohamed Alaa Nady

**Affiliations:** 1 Cardiothoracic Surgery Department, Assiut University Heart Hospital, Assiut, Egypt; 2 Cardiothoracic Surgery, Assiut University Heart Hospital, Assiut, Egypt

**Keywords:** Sternum, Fracture, Reconstruction, Titanium, Plates, Screws, Pain

## Abstract

**Background:**

Sternal fractures are rare accounting for about 3–8% of traumatic chest. There are many lines of treatments for sternal fractures which can be classified as conservative or surgical. Surgical techniques include wire fixation and sternal plating. There are no standardization of indications for each line of management.

We explore if sternal reconstruction using locking titanium plates and self-tapping screws provide the patient with the best chance of proper sternal healing avoiding chronic pain and its complications and allow the patient early mobilization and rapid restoring of his normal life at its maximum.

**Methods:**

Our inclusion criteria are patients of both gender from 20 to 60 years of age presented with traumatic sternal fracture at any site or pathological fracture due to metastatic or primary tumors infiltrating the sternum. High Associated Injury Scale scores were excluded. Exclusion criteria also included patients younger than 20 years or older than 60 years. Primary outcome is post-operative pain score and is measured using numerical pain scale ranging from zero to 10 where zero means there is no pain at all and ten is the worst imaginable pain ever.

**Results:**

Sternal reconstruction using titanium plates has proven to be an efficient method of stabilization with tremendous immediate relief of pain showed by the differences between pre-operative and post-operative pain scale scores in our patients (*n* = 5) with Median scores being 7 and 1.5 with range being from 7 to 9 and 1 to 3 respectively (*p*-value = .039). Operative time range between 150 min and 90 min with median of 120 min. Extubation of patients was smooth with no events with median time of extubation being 120 min. From our experience, there were no observed wound complications except at the third patient who suffered a small wound hematoma that was resolved by gentle compressing only and needed no further intervention.

**Conclusion:**

We recommend adopting sternal reconstruction using titanium plating systems more readily encouraging even larger clinical trials on the way to a clear guidelines. Plating systems show promising results with least pain, better stability, less complications and rapid, smooth recovery.

**Trial registration:**

NCT04092374

## Background

Sternal fractures are rare accounting for about 3–8% of traumatic chest injuries [1, 2]. It needs high velocity trauma especially common with deceleration accidents. There are many lines of treatments for sternal fractures which can be classified as conservative or surgical. Surgical techniques include wire fixation and sternal plating. There are no standardization of indications for each line of management [2].

We explore if sternal reconstruction using locking titanium plates and self-tapping screws provide the patient with the best chance of proper sternal healing avoiding chronic pain and its complications and allow the patient early mobilization and rapid restoring of his normal life at its maximum especially for those patients who depend on active lifestyle.

## Methods

This is a case series of patients who needed sternal reconstruction for traumatic and pathological causes. Our inclusion criteria are patients of both gender from 20 to 60 years of age presented with traumatic sternal fracture at any site or pathological fracture due to metastatic or primary tumors infiltrating the sternum. High Associated Injury Scale scores were excluded due to affection on our primary outcome acting as a confounder. Exclusion criteria also included patients younger than 20 years or older than 60 years. Primary outcome is post-operative pain score and is measured using numerical pain scale ranging from zero to 10 where zero means there is no pain at all and ten is the worst imaginable pain ever. Our secondary outcomes include hospital stay and period of bed recumbence. Sternal fracture is defined as discontinuity of sternal cortex at any part of the cortex palpated on examination and seen by imaging techniques such as CXR and CT chest. Planning is done based on prior assessment of patients’ general condition, their preferences and a written consent is signed. Choosing type and shape of plates is made based on site of fracture and mechanics of movement at this part to provide the most achievable stability. End state variables will be designated, such as, morbidity, or mortality. Morbidity will include wound infection, postoperative pain, delayed healing, sternal dehiscence & postoperative length of hospital stay at short term post-operative period. Analysis of post-operative pain will be done using numerical pain score system. Post-Operative sternal healing will be assessed by the standard suitable imaging modality, MSCT Chest with 3D Reconstruction is used in all casesWound healing will be assessed by close monitoring for signs of inflammation or infections such as redness, increasing pain, wound discharge or swelling.

Titanium plates were used with different shapes and sizes according to site of fracture from BIOMET™ with 2.4*8 mm self-tapping screw.

## Results

### First case

**Fig. 1:**
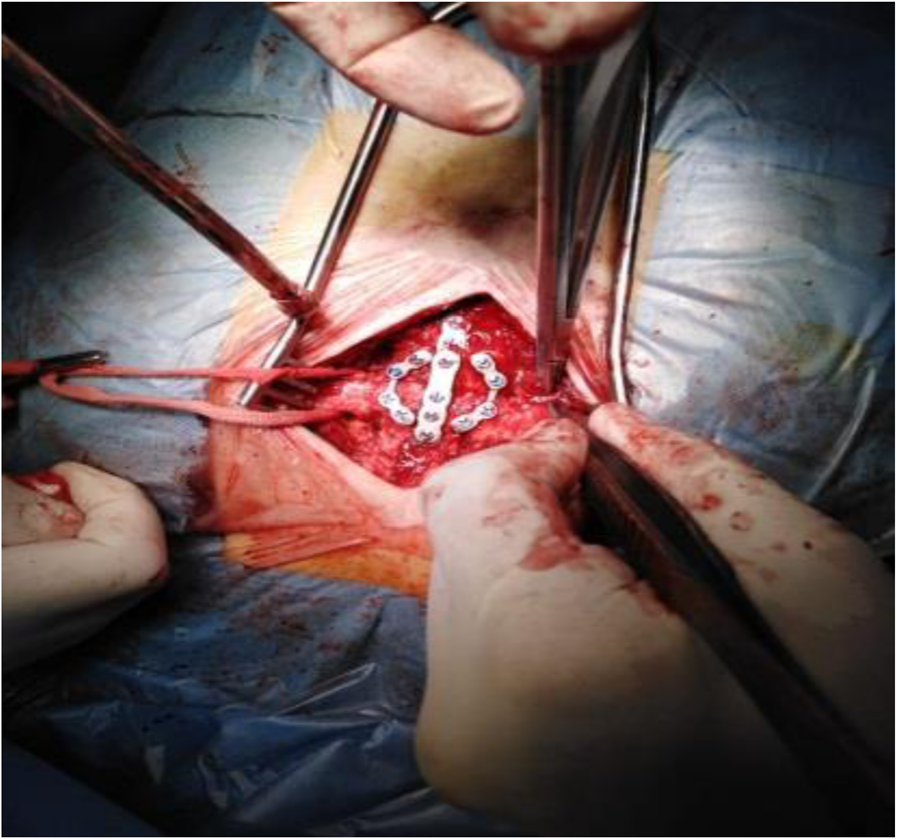
Intra-operative Plates in situ

**Fig. 2:**
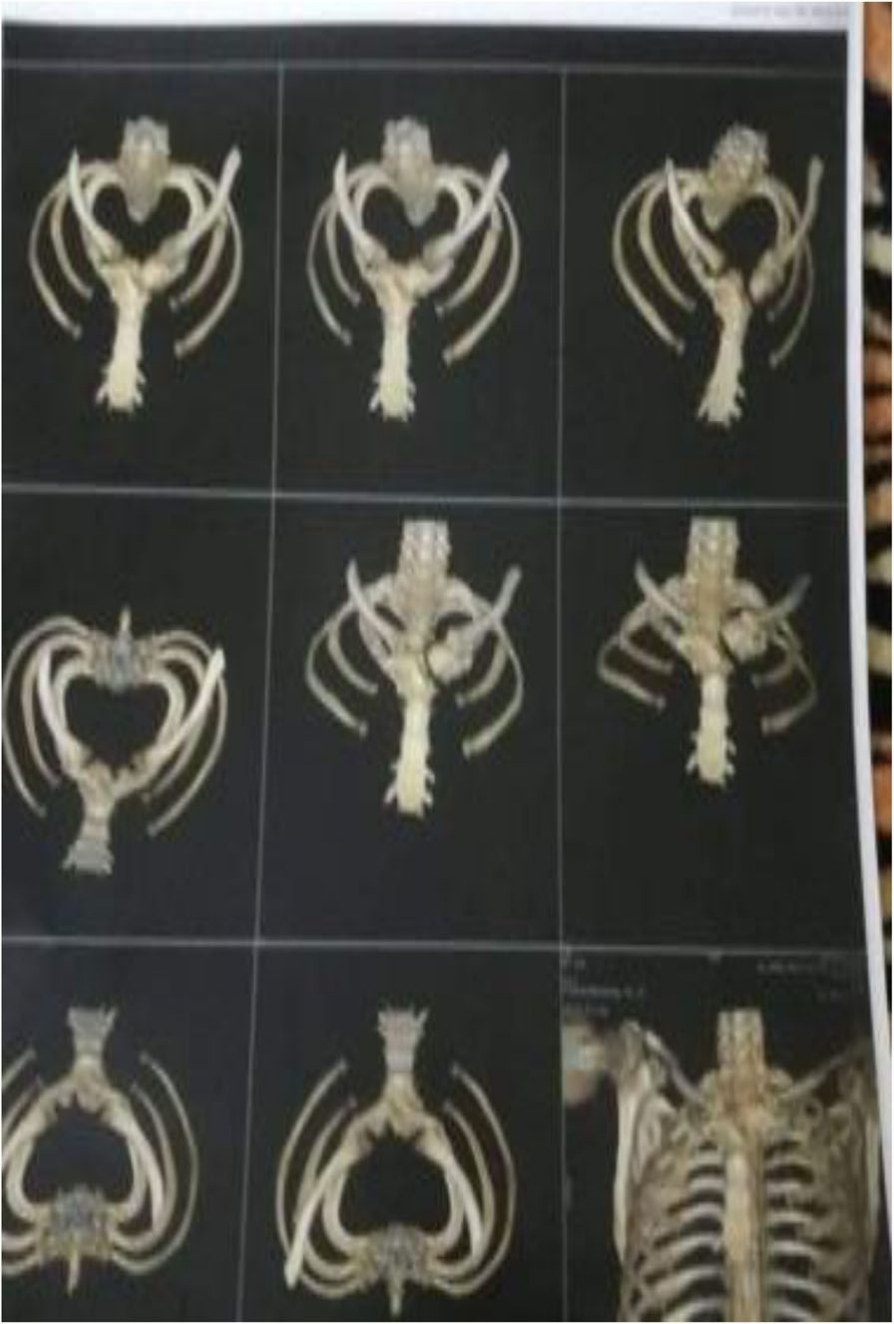
Pre-operative MSCT chest showing fractures sites

Male patient 45 years old, presented to trauma department with motor vehicle accident (MVA), general condition was good with GCS 14, vitally stable. Examination showed bruising on chest with tenderness over upper chest. Primary survey showed suspicious appearance at manubrium at CXR so we proceeded with MSCT chest for further evaluation and it showed fracture of manubrium together with left sterno-clavicular joint fracture and no other abnormalities were detected. Planning for surgery is done after counselling the patient. We used 4-holes straight titanium plate for manubrial fracture and 4-holes orbital titanium plate with titanium 2.7 mm auto self-tapping screws for further stabilization at manubrio-sternal joint. Post-operative pain score was 1 compared with 7 pre-operatively {*P*-value (.034)} with early recovery and ambulation. Hospital stay was 2 days including the day of surgery. Early Post-operative follow-up showed excellent wound healing with no seroma nor surgical site infection (Figs. 1 and 2).

### Second case

Male patient, 50 years old presented to trauma department with blunt trauma to chest because of motor vehicle accident. Patient was vitally stable, GCS 15 with agonizing pain. Appropriate analgesia is administrated together with primary survey which showed mid-sternal fracture at lateral CXR and no other abnormalities could be detected. MSCT chest was obtained for further evaluation of the fracture. It showed mid-sternal fracture. Counselling of the patient with planning for surgery is made. One orbital titanium plate is used for fixation of the fracture. Post-operative pain score was 1. Hospital stay was 2 days with early mobility and rapid recovery without any respiratory function complications.

### Third case

**Fig. 3:**
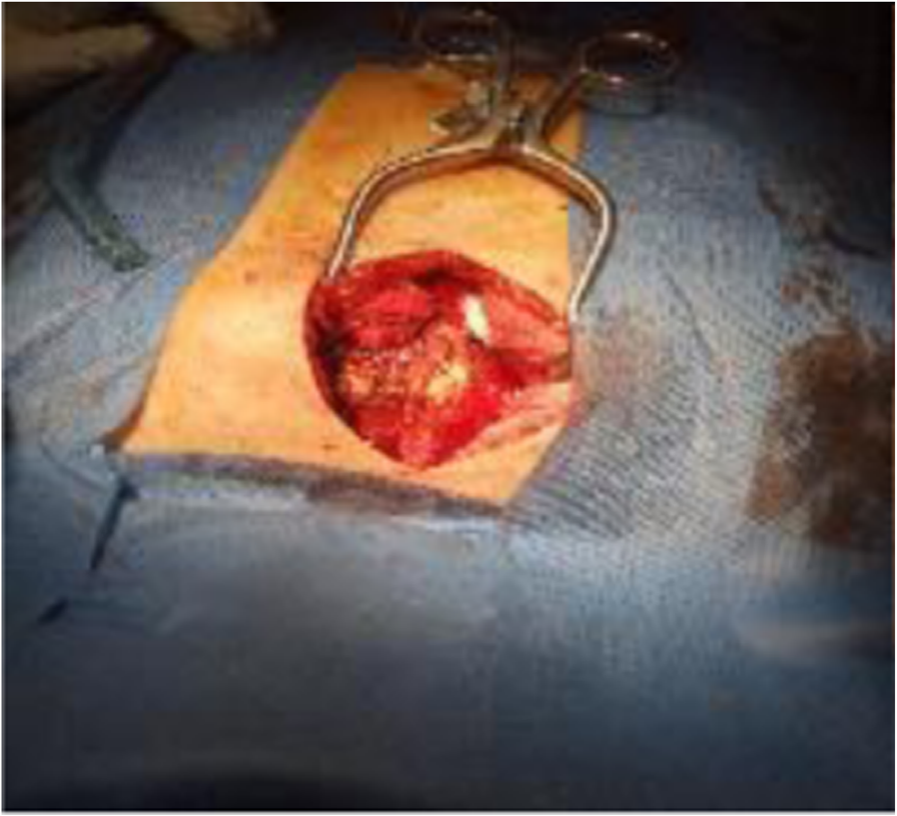
Preoperative photo showing fracture at manubrio-sternal joint

**Fig. 4:**
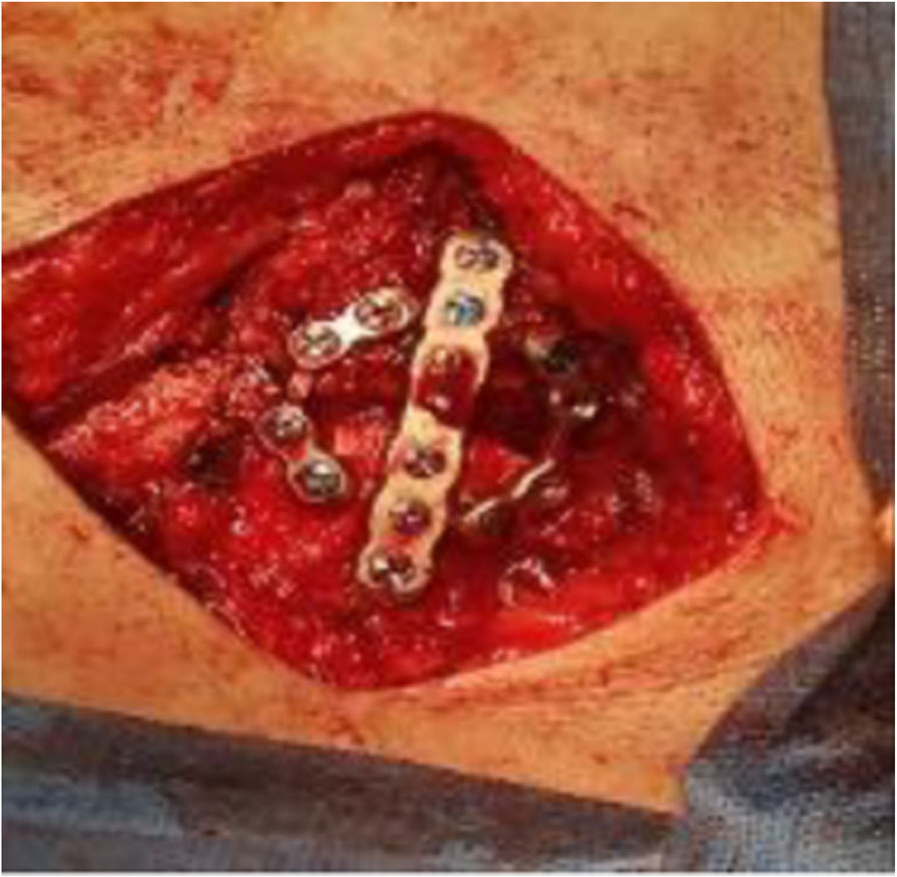
Intraoperative fixation of sternal fracture by one straight and 2 orbital titanium plates

**Fig. 5:**
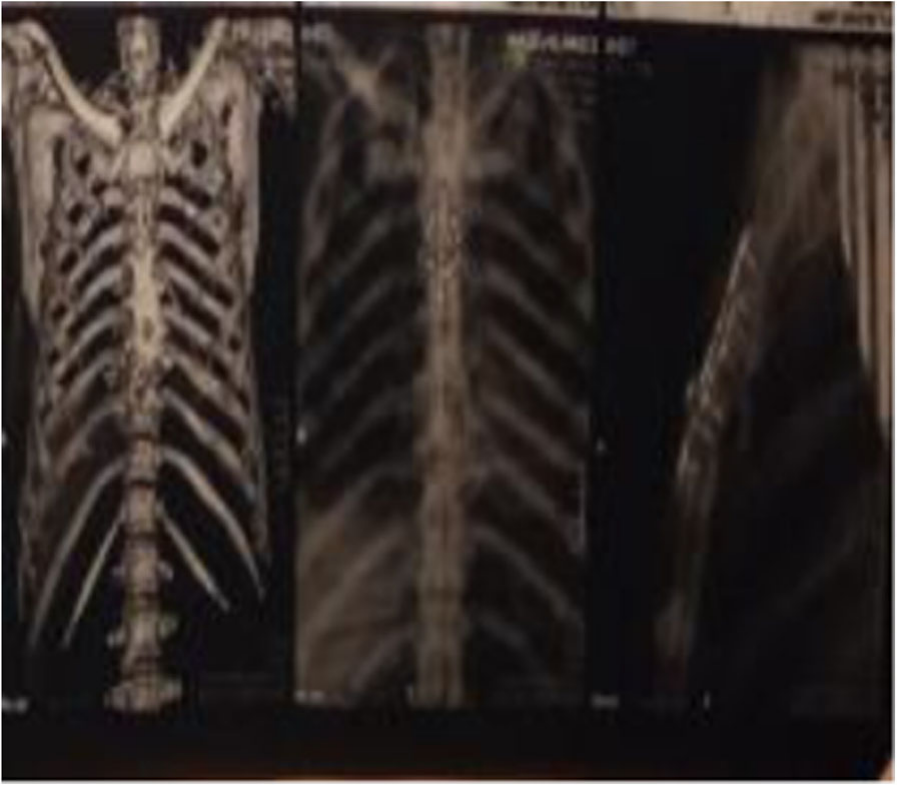
Postoperative MSCT chest showing Titanium plates in position

Male patient, 45 years presented to our trauma department with motor vehicle accident, GCS could not be assessed due to motor system affection. Patient presented vitally stable with BP 110/70, HR 85, SaO2 on Room air (RA) 97%. Primary survey showed abnormal sternal appearance on CXR together right radial fracture. MSCT chest showed manubrio-sternal joint fracture with mild lung contusion. MSCT spine showed cervical injury and patient needed collar for stabilization of cervical spine. Patient was unable to move both his legs and showed sensory level affection starting at T 10 dermatome (umbilicus). His right forearm was put in plaster of Pairs (POP) after orthopedics evaluation. Counselling of patient is done about options of fixation of his sternal fracture and planning for plate fixation is made after his choice and consenting. Although this patient has high associated injury scale (AIS) score, we were concerning about providing our patient with best options for better coping and faster recovery from his sternal fracture which was adding sever pain to his suffering. We used straight titanium plate with two orbital plates at each side for better stability of his sternum and pain recovery. His pain score was not reliable due to other confounding factors which are cervical agonizing pain and his sensory level loss. However, he showed excellent recovery post-operatively in the form of good wound healing, normal respiratory function tests without any deterioration which gives us a clue about stability and recovery of his sternum that caused no further pain which if persisted could lead to chest stitching pain with respiration leading to decrease inspiratory reserve volume, atelectasis and suppression of cough mechanisms with chest infections super-imposed. Hospital stay was 40 days due to orthopedics and neurosurgery follow-up (Figs. 3, 4 and 5).

### Fourth case

**Fig. 6:**
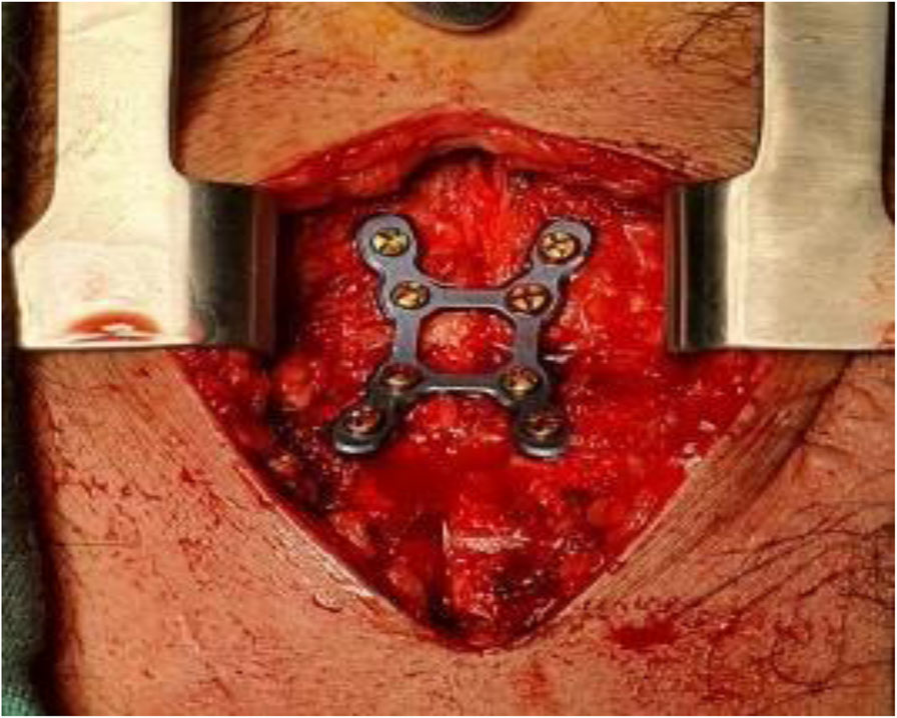
Intraoperative H-shaped plate in position

Male patient, 55 years, presented with blunt trauma to his chest of high velocity. Primary survey of the patient showed no abnormalities. Sever tenderness on his chest raised our suspicious so we proceeded with MSCT chest. It showed fracture at his manubrium. We used H-shaped titanium plate. His Pain score was 2. His hospital stay was 4 days (Fig. 6).

### Fifth case

Male patient, 42 years old, was diagnosed with chondrosarcoma at the body of the tumor. MSCT chest is done for staging and evaluation of the patient alluding its suitability for excision. After discussion with patient and consenting, surgical excision was the decision. At exploration the mass had infiltrated the whole body of the sternum and overlying pectoral muscle. Surgical excision of the body of the sternum together with the overlying pectoralis is done leaving a big gap. Titanium mesh 20*10 cm is used fixed by 2 mm titanium screws and sandwiched between 2 layers of prolene meshes. Post-operative pain score was 2 at third day and he was discharged successfully at that day with good recovery. At 1 week post-operative follow-up, there has been good stability of chest wall with perfect wound healing (Table 1).

**Table 1:** Table shows summary of different variables of our represented cases with means of values

	Case 1	Case 2	Case 3	Case 4	Case 5	Median
Age (years)	45	50	45	55	42	45
Pain Score (Post-Operative)	1	1	Unreliable^a^	2	2	1.5
Operative Time (minutes)	120	100	90	130	150	120
Ex-tubation (minutes)	120	120	60	120	120	120
Hospital Stay (Days)	2	2	40^b^	3	2	N\A^b^

^a^Pain score for third case is unreliable due to High Associated injury scale (AIS) score. Patient suffered from spinal trauma with sensory loss at level T10 dermatome with cervical vertebrae injury

^b^Increased length of hospital stay is due to associated injuries

## Discussion

Sternal reconstruction using titanium plates has proven to be an efficient method of stabilization with tremendous immediate relief of pain showed by the differences between pre-operative and post-operative pain scale scores in our patients (*n* = 5) with Median scores being 7 and 1.5 with range being from 7 to 9 and 1 to 3 respectively (*p*-value = .039). Operative time range between 150 min and 90 min with median of 120 min. Extubation of patients was smooth with no events with median time of extubation being 120 min. From our experience, there were no observed wound complications except at the third patient who suffered a small wound hematoma that was resolved by gentle compressing only and needed no further intervention. Indeed, wound seroma or hematoma was reported in many literature with a rate of 24% of cases [3, 4] and there were no relations between its incidence and using plating systems [4, 5]. Instead, It can be better explained by hemostasis issues or pectoral vessels minors bleeds [6]. Bleeding was reported and is most probably due to intercostal vessels injury [7]. This might have higher incidence with transverse plates and reckless drilling or inappropriate length of screws. However, this should be least likely to occur with longitudinal plates [8]. We recommend cautious evaluation of the depth of drilling and length of the screws to be used. Post-operative analgesia for all our patients is ibuprofen tabs 400 mg PO twice daily for 5 days. Prior to extubation and after transfer from OR to ICU, ketorolac 30 mg IV bolus is given to all our patients once.

Plating stabilization of sternum shows mechanical superiority over other methods of stabilization taking into consideration the liability of this part of chest wall to motion with spontaneous ventilation and minor movement being an important point of muscle and ribs attachment and the complex mechanics of chest wall. Trivial movements are making shear forces which disrupt healing process needing absolute stability necessitating good stabilization [9, 10]. This gives plating technique the advantage of providing better and faster sternal healing keeping respiratory functions unaffected with normal chest wall motion avoiding atelectasis and chest infections resulting from severe pain affecting respiration [11, 12].

Small number of cases [13–15] presented in our work hinder us incapable of giving a strict recommendations yet we think that our results are promising and sharing such results with the international scientific community will help encouraging more surgeons to explore this new technique. We are hoping that our upcoming work will be presented with more cases enough to reach clear guidelines on using plating technique for sternal reconstruction.

## Conclusion

We recommend exploring sternal reconstruction using titanium plating systems more readily encouraging even larger studies on the way to a clear guidelines. Giving our patients the best available curative options for sternal reconstruction with better Quality of Adjusted Life Years (QALY) should be our main consideration. Plating systems show promising results with least pain, better stability, less complications and rapid, smooth recovery.

## Data Availability

The datasets used and/or analyzed during the current study are available from the corresponding author on reasonable request.

## Abbreviations

LCP: Locking Compression Plates
NSAIDs: Non-Steroidal Anti-Inflammatory Drugs
CXR: chest X-Rays
MSCT: Multi Slice Computed Tomography
POP: Plaster Of Paris
GCS: Glasgow Coma Scale
QAYL: Quality of Adjusted Years of Life
AIS: Associated Injury Scale
